# High proportions of *pfhrp2* gene deletion and performance of HRP2-based rapid diagnostic test in *Plasmodium falciparum* field isolates of Odisha

**DOI:** 10.1186/s12936-018-2502-3

**Published:** 2018-10-29

**Authors:** Pallabi Pati, Gunanidhi Dhangadamajhi, Madhusmita Bal, Manoranjan Ranjit

**Affiliations:** 1 Molecular Epidemiology Laboratory, ICMR-Regional Medical Research Centre, Bhubaneswar, 751023 India; 2 Department of Biotechnology, North Orissa University, Baripada, Odisha 757003 India; 3 Immunology Laboratory, ICMR-Regional Medical Research Centre, Bhubaneswar, 751023 India

**Keywords:** *Plasmodium falciparum*, HRP-2, RDT, *pfhrp2*, *pfhrp3*, Genotyping, Odisha, Season

## Abstract

**Background:**

With the documentation of cases of *falciparum* malaria negative by rapid diagnostic tests (RDT), though at low frequency from natural isolates in a small pocket of Odisha, it became absolutely necessary to investigate the status of HRP-2 based RDT throughout the state and in different seasons of the year.

**Methods:**

Suspected individuals were screened for malaria infection by microscopy and RDT in 25/30 districts of Odisha, India. Discrepancies in results were confirmed by PCR. False negative RDT samples for *Plasmodium falciparum* mono-infection were evaluated for detection of HRP2 antigen in ELISA and genotyped for *pfhrp2*, *pfhrp3* and their flanking genes. Multiplicity of infection was ascertained based on *msp1* and *msp2* genotyping and parasitaemia level was determined by microscopy.

**Results:**

Of the total 1058 patients suspected for malaria, 384 were microscopically confirmed for *P. falciparum* mono-infection and RDT failure was observed in 58 samples at varying proportion in different regions of the state. The failure in detection was due to undetectable level of HRP-2. Although most of these samples were screened during rainy season (45/345), significantly high proportion (9/17) of RDT negative samples were obtained during the summer compared to rainy season (P = 0.0002; OR = 7.5). PCR genotyping of *pfhrp2* and *pfhrp3* in RDT negative samples showed 38/58 (65.5) samples to be *pfhrp2* negative and 24/58 (41.4) to be *pfhrp3* negative including dual negative in 17/58 (29.3). Most of the RDT negative samples (39/58) were with single genotype infection and high proportions of *pfhrp2* deletion (7/9) was observed in summer. No difference in parasitaemia level was observed between RDT positive and RDT negative patients.

**Conclusion:**

High prevalence of parasites with *pfhrp2* deletion including dual deletions (*pfhrp2* and *pfhrp3*) is a serious cause of concern, as these patients could not be given a correct diagnosis and treatment. Therefore, HRP2-based RDT for diagnosing *P. falciparum* infection in Odisha is non-reliable and must be performed in addition to or replaced by other appropriate diagnostic tools for clinical management of the disease.

**Electronic supplementary material:**

The online version of this article (10.1186/s12936-018-2502-3) contains supplementary material, which is available to authorized users.

## Background

With the recommendation of the World Health Organization (WHO) that only parasitologically confirmed cases of malaria patients should be treated with appropriate anti-malarial drugs [1], simple, reliable and species specific diagnostic methods for detecting malaria infections became absolutely necessary. Clinical diagnosis being unreliable and non-specific, it required confirmation through parasitological diagnosis which involved identification of parasites by their direct observation in patient’s blood [such as microscopy and quantitative buffy coat (QBC) test], through identifying parasite nucleic acids by molecular methods [such as PCR, loop-mediated isothermal amplification (LAMP) method, microarray] or parasite specific antigens/antibody by rapid diagnostic tests and other new strategies (such as mass spectrometry and flow cytometry). However, each of these methods had their own limitations and many of these tests are non-amenable for point-of-care diagnosis, particularly in resource-limited endemic regions.

Although microscopy served as the gold standard for malaria diagnosis, given its unavailability in resource-poor settings and requirement of technical expertise [2], rapid diagnostic tests (RDTs) became the widely adopted alternative choice as onsite test for its ease to perform, comparable sensitivity with microscopy, not requiring electricity and being quick [2–4].

Detection of malaria by RDTs are principally based on identification of one or more of the three antigens, such as histidine-rich protein-2 (HRP2), lactate dehydrogenase (LDH), and aldolase [5, 6]. Of these, HRP2 is used for specific detection of *Plasmodium falciparum* because of its exclusive expression in this species of human *Plasmodium* at asexual and sexual phases in the blood stage infection [7–10], while LDH and aldolase are pan-specific as these are produced by all human malaria parasites. Although LDH can also be used for specific detection of *P. falciparum* infection, its sensitivity is less compared to HRP2 and, therefore, HRP2 has been used in almost all of the RDTs for *P. falciparum* detection [11]. However, increasing reports on variable test performances of same HRP2-based RDT in different endemic regions and different tests on panels of blood samples targeting PfHRP2 [12] is of concern, and has been attributed to device-related factors (such as quality of manufacture, storage condition, techniques for carrying out the test and interpretation of the test results) [13–15] and parasite factors (such as parasite density, quantity of parasite antigen produced or its persistence in peripheral blood and variability of target epitopes in antigen structure) [11, 16, 17]. Of these, the most important parasite factors of the observed variability in sensitivities in recent years have been confined to lack of the *pfhrp2* gene in the parasite species resulting in no expression of the corresponding antigen [17–22] or variability of target epitopes (its presence or absence and copy number variation) within the PfHRP2 antigen due to genetic diversity in the gene [22–27]. Besides, the PfHRP3 antigen which shares structural similarities to some extent with PfHRP2 has been thought to cross-react with PfHRP2 antibody [12] and may influence the diagnostic performance of PfHRP2-detecting malaria RDTs. While *pfhrp2* gene is located on subtelomeric region of chromosome 7 flanked by a pseudogene (PF3D7_0831900/MAL7P1.230) and a putative heat shock protein 70 gene (PF3D7_0831700/MAL7P1.228), *pfhrp3* is located on subtelomeric region of chromosome 13 and is immediately flanked by a gene of unknown function, (PF3D7_13721000/MAL13P1.485) in the upstream and a gene for acyl-CoA synthetase (PF3D7_1372400/MAL13P1.475) in the downstream. Both PfHRP2 and HRP3 share many structural similarities with signal peptide sequence located in both on exon 1, and exon 2 harbours histidine (H) and alanine (A) rich amino acid repeats [20].

Since, malaria in Odisha is largely due to *P. falciparum* infections (> 85%) followed by *P. vivax* and *P. malariae* [28], HRP2-based RDT along with pan-specific LDH or aldolase had been considered the best choice for malaria diagnosis. Further, in Odisha, India, the majority of severe malaria and malaria related deaths are ascribed to *P. falciparum* infections with few instances of severe malaria were due to *P. vivax* [29]. In such cases, failure of HRP-2 based RDT could hamper the early diagnosis and case management of severe patients which may largely affect the malaria control. Until now, except one study [30] documenting the low prevalence of RDT negative *P. falciparum* isolates from a small pocket of Odisha, the status of HRP-2 based RDT as a reliable and accurate method of diagnosing malaria in Odisha has not been evaluated systematically. It is unknown whether there exists genetic diversity in the *pfhrp2* or *pfhrp3* genes affecting HRP-2 detection of *P. falciparum* infection throughout the state of Odisha or whether significant proportion of parasites are still capable of expressing the HRP2 antigens. In this paper, failure of HRP-2 based RDT in detecting *P. falciparum* infections concomitant to gene deletion and genetic diversity in *hrp2/hrp3* genes are reported among clinical isolates across geographically diverse region of Odisha collected during different seasons. The information generated in this study would be useful for guiding malaria control strategies in this endemic region by providing clue for appropriate incorporation of RDTs in the management of malaria.

## Methods

### Sample collection and diagnosis of malaria

**Fig. 1:**
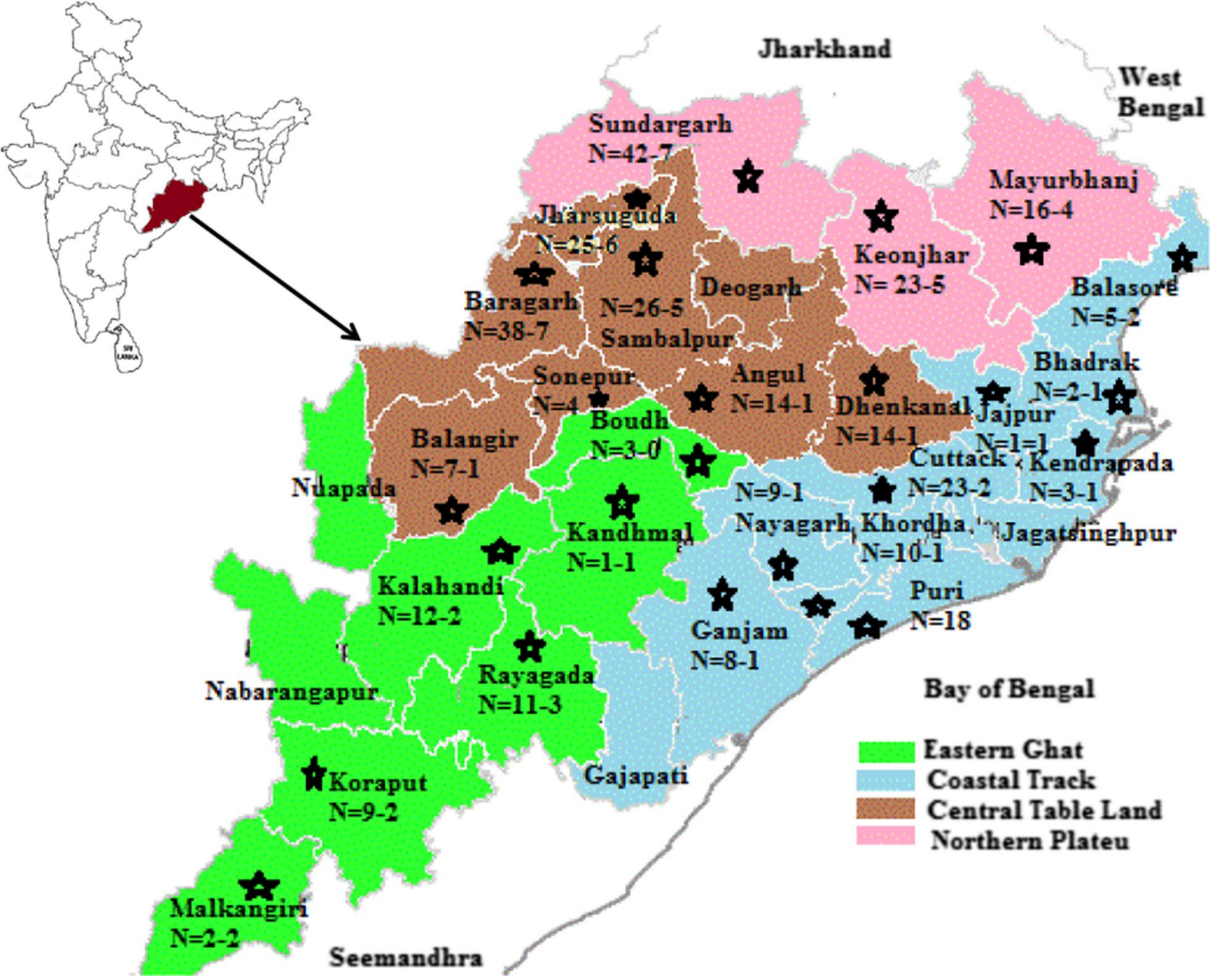
Site of sample collections and distribution of RDT negative parasite isolates in Odisha, India

The present cross-sectional study was conducted during 2013–2016 mostly on the peak season of malaria transmission (July to October, rainy season) and performed in 25/30 districts representing all four geo-physically different topological areas of the state of Odisha, India (Fig. 1). Occasional samplings were also done during summer and winter. About 1 ml of blood samples were obtained in ethylene-diamine-tetra-acetic acid (EDTA) containing vials from uncomplicated febrile patients suspected for malaria infections of 5 years or older (excluding pregnant women) age after their willingness and written consent to participate in the study. In the case of children, parents or guardians (wherever necessary) signed written consent form prior to sample collection. All blood samples were tested for the presence of malaria infection using HRP-2 based RDT (SD Bioline Malaria Ag Pf) in the field before the expiry period. For samples showing negative results by SD Bioline RDT in field, tests were performed twice (one with SD Bioline and another with Carestart™ Malaria HRP2 Pf) in laboratory and waited for at least 15–30 min to obtain proper result. Giemsa-stained (thick and thin) blood smears of these samples were also prepared and transported with care for examination by microscopy at Regional Medical Research Centre, Bhubaneswar, Odisha. Blood slides were declared negative for malaria infections when no parasites were seen in about 100 thick film fields and the RDT were performed as per manufacturer’s instructions. In order to ensure the quality of microscopy diagnosis of *P. falciparum* malaria, the independent diagnostic performance results of one author and a laboratory technician was cross examined by another author and the consistently accurate results were considered for malaria infection. In cases of discrepancy of results by both microscopy and RDT, species-specific identification of malaria parasite by PCR amplification was employed for confirmation. Only, mono-infected *P. falciparum* samples were enrolled for further analysis in the study and patients with severe complication, co-infection with other *Plasmodium* species or chronic infections with malaria were excluded from analysis.

### Determination of parasitaemia

Only *P. falciparum* infected blood slides were examined for determination of asexual blood parasitaemia/µl with the assumption that approximately 8000 number of white blood cells (WBC) was present per microlitre of blood. The parasite densities were calculated as number of parasites/μl = number of asexual parasites/200 WBCs × 8000.

### Detection of HRP2 protein using ELISA

For samples negative for HRP-2 based RDT but positive for microscopy and PCR, ELISA based detection of PfHRP-2 was performed to cross-check the HRP-2 negativity using the Malaria Ag CELISA kit (CELLABS Pty, Sydney, Australia) as per manufacturer’s instructions. Quantification of PfHRP-2 was done by plotting absorbance values against a standard curve of recombinant HRP2 which is considered as the standard procedure for quantification in ELISA [31].

### Parasite DNA extraction, PCR analysis and DNA sequencing

The parasite genomic DNA was purified from 100 μl of blood following the standard protocol [32] with little modification as established in authors’ laboratory previously [33]. In brief, blood cells were lysed with lysis buffer (10 mM Tris-HCI, pH 8.0; 0.1 M EDTA, pH 8.0; 0.5% SDS, and 20 μg/ml pancreatic RNase) at 37 °C for 2 h and then treated with proteinase K (100 μg/ml) followed by overnight incubation at 54 °C. On the next day, DNA was obtained by phenol–chloroform extraction and isopropanol precipitation. Upon air-dry of precipitated DNA, it was then resuspended in 50 μl of DNase free water and stored at − 20 °C. The extracted DNA was used for amplification of the parasite DNA in separate reactions with each of the RDT negative but microscopy positive samples in a final reaction mixture of 20 µl using species specific PCR primers as described by Snounou et al. [34] for confirmation of *P. falciparum* mono-infection. Besides, the *P. falciparum* merozoite surface protein 1 (*pfmsp1*), and 2 (*pfmsp2*) were amplified to assess the DNA quality and for determining multiplicity of infection based on previous description [30, 33, 35] in these RDT negative samples. DNA samples which showed more than one fragment for a locus in PCR amplification was considered to be infected with two or more genetically distinct parasite strains or multiple genotype infection (MGI). PCR detection for the presence of *pfhrp2* and *pfhrp3* genes and their genetic diversity in terms of amino acid repeat sequences was carried out by amplifying exon 2 of both genes following the primers and amplification procedure described in Baker et al. [36] and subsequent sequencing of amplified DNA. Moreover, flanking genes for *pfhrp2* and *pfhrp3* were amplified based on the primer sequences and procedure of amplification as described in Abdallah et al. [20]. Failures of amplification in first attempt of PCR were re-tested for possible polymorphisms at the primer binding site by lowering the annealing temperatures. None of the cases amplification was successful except for positive control indicating PCR failure was due to gene deletion. Besides, a different set of primers were used [24], when failure in amplification of *pfhrp2* and *pfhrp3* were observed for further confirmation of gene deletion. The primer sequences and thermo-cycling conditions for amplification of different genes are described in Additional file 1: Table S1.

Gene deletion of *pfhrp2* or *pfhrp3* was declared when the samples showed positive amplifications for *msp1*, *msp2* and positive control (clinical isolate positive for all loci under the study) but negative for either *hrp2* or *hrp3*. DNA sequencing was performed for positive products of *pfhrp2* and *pfhrp3* genes from both directions using forward and reverse primers of exon 2. PCR products were purified by using spin columns (Qia Quick Gel extraction kit, Germany,) as per recommended protocol of manufacturer and used in a standard dye terminator (BigDye Terminator v3.1 Cycle Sequencing Kit) DNA sequencing on an Applied Biosystems 3130 XL sequencer.

### Statistical analysis

Statistical analysis was performed using GraphPad Prism 5.0 (GraphPad Software Inc., USA) and SPSS version 23 (IBM, Armonk, NY, USA). Differences in frequency distribution of RDT negative isolates from different regions of the state and seasons of the year were tested by χ^2^ test computing odds ratio and 95% confidence interval. Mean parasite densities between two groups was compared using Mann–Whitney tests, whereas Kruskal–Wallis test with Dunn’s multiple comparison was used when three or more groups (RDT positive, RDT negative, double negative for *hrp2/hrp3*, double positive for *hrp2/hrp3*) were involved. A P-value of < 0.05 was considered as significant for all statistical analysis.

## Results

**Fig. 2:**
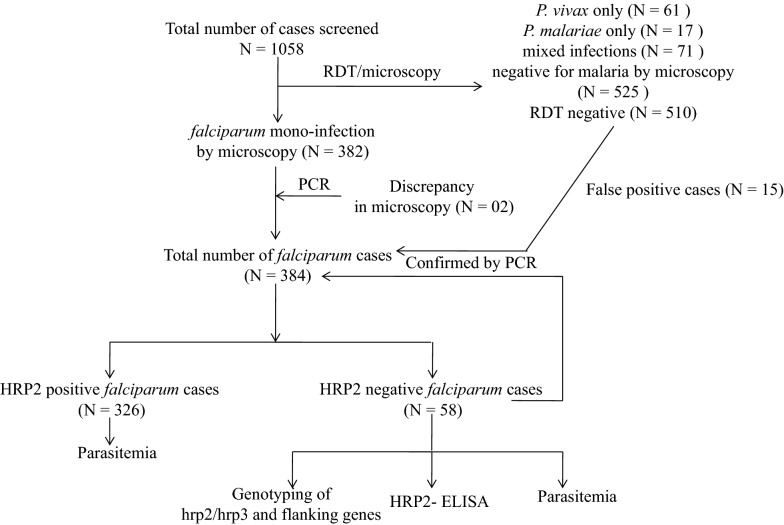
Flow chart of sampling and different tests applied in the study

Of the total 1058 malaria suspected patients, 384 (36.3%) samples met the inclusion criteria by yielding positive results of mono-infection by *P. falciparum* and considered for further analysis by RDT (Fig. 2). About 84.9% of samples diagnosed positive for *P. falciparum* mono-infection by microscopy were also found positive by RDT, whereas RDT failure was detected in 15.1% (58) of the cases. The sensitivity and specificity of RDT (SD Bioline) was found to be 84.9% and 97.14% respectively. Although most of the samples were screened and collected during rainy season (Table 1), significantly high proportion of (52.9%) RDT negative samples were obtained during the summer (P = 0.0002; OR = 7.5, 95% CI 2.75–20.45) compared to rainy season. Region wise distribution of RDT negative results showed high frequency of RDT failure in eastern ghat (EG) region followed by northern plateau (NP), whereas coastal tract (CT) and central table land (CTL) had comparable negative results. About 67.2% of RDT negative result was due to single genotype infection of the parasite strain. No difference in RDT diagnosis was obtained with regard to gender.

**Table 1:** Prevalence of RDT negative samples along with gene deletions at *pfhrp2* and *pfhrp3* in different region and seasons of the year

Parameters	Samples screened	Positive for *P. falciparum*^a^	HRP2 negative	Gene deletion in *Pfhrp2* and *hrp3* in HRP2 negative samples					
				PfHRP2		PfHRP3		PfHRP2 and PfHRP3	
				Positive	Negative	Positive	Negative	Positive	Negative
Geophysical region									
Northern plateau	289	97	16 (16.5)	07 (12.1)	09 (15.5)	10 (17.2)	06 (10.3)	04	03 (5.2)
Coastal tract	243	76	10 (13.15)	04 (6.9)	06 (10.3)	07 (12.1)	03 (5.2)	03 (5.2)	02 (3.4)
Central table land	409	163	22 (13.5)	06 (10.3)	16 (27.6)	08 (13.8)	14 (24.1)	03 (5.2)	11 (19)
Eastern ghat	117	48	10 (20.83)	03 (5.2)	07 (12.1)	09 (15.5)	01 (1.7)	03 (5.2)	01 (1.72)
Season									
Summer	79	17	09 (52.94)	02 (3.4)	07 (12.1)	06 (10.3)	03 (5.2)	02 (3.4)	03 (5.2)
Rainy	767	345	45 (13.04)	16 (27.6)	29 (50)	25 (43.1)	20 (34.5)	09 (15.5)	13 (22.4)
Winter	212	22	04 (18.18)	02 (3.4)	02 (3.4)	03 (5.2)	01 (1.7)	02 (3.4)	01 (1.72)
Gender									
Male	672	299	44 (14.71)	18 (31)	26 (44.8)	29 (50)	15 (25.9)	13 (22.4)	10 (17.2)
Female	386	85	14 (16.47)	02 (3.4)	12 (20.7)	05 (8.6)	09 (15.5)	0	07 (12.1)
Multiplicity of infections									
SGI	–	ND	39 (67.2)	12 (20.7)	27 (46.6)	24 (41.4)	15 (25.9)	10 (17.2)	13 (22.4)
MGI	–	ND	19 (32.8)	08 (13.8)	11 (19)	10 (17.2)	09 (15.5)	03 (5.2)	04 (6.9)
Total	1058	384	58 (15.1)	20 (34.5)	38 (65.5)	34 (58.6)	24 (41.4)	13 (22.4)	17 (29.31)

Value within the parentheses for HRP2 negative are percentage value derived from total number of *P. falciparum* positive samples in each region, season or gender, whereas for gene deletion percentage values were calculated from total number of 58 samples negative by RDT

*ND* Not determined for RDT positive samples, only RDT negative samples were screened for *msp1* and *msp2* typing for quality check of DNA and multiple genotype infections

^a^Mono infected *Plasmodium falciparum* cases by microscopy

### PCR genotyping and haplotype pattern

In a total of 58 RDT negative samples, PCR genotyping of *hrp2/hrp3* gene along with their flanking genes amplification were carried out. Of which, 38 (65.5) samples had *hrp2* negative and 24 (41.4) had *hrp3* negative including 17 (29.31) samples double negative at both the loci (Table 1). Most of these *hrp2* and *hrp3* deletions were observed in SGI of *P. falciparum* and in samples collected during rainy season. However, high proportions of *hrp2* negative samples were obtained during the summer (7/9), whereas *hrp3* negative (20/45) samples were obtained during the rainy season, mostly from CTL region (16/22 and 14/22, for *hrp2* and *hrp3*, respectively). Genotyping of *msp1* and *msp2* genes in all RDT negative isolates revealed single genotype infection (SGI) in 39 (67.2%) samples, which were used to construct haplotype pattern based on absence and presence of *hrp2/hrp3* and their flanking genes. A total of 10 different patterns were observed as shown in Table 2. Of these, pattern 8 and 10 were observed at higher proportion and had presence of all loci under consideration except for *hrp2* in pattern 8 (Table 2). These were followed by pattern 7 and 1, both of which were double negative for *hrp2* and *hrp3*. Deletion of flanking genes for both *hrp2* and *hrp3* were less frequent in this study.

**Table 2:** Frequency distribution of haplotypes based on presence and absence of *hrp2*, *hrp3* and their flanking genes along with associated parasite density

Pattern	hrp2F1	hrp2	hrp2F2	hrp3F1	hrp3	hrp3F2	Frequency	Percent	Parasite density
P1	−	−	+	+	−	+	4	10.3	1877 ± 3221
P2	−	+	−	+	−	+	1	2.6	383 ± 0.0
P3	+	−	−	−	+	+	1	2.6	157 ± 0.0
P4	+	−	−	+	+	+	2	5.1	5421 ± 806.1
P5	+	−	+	−	−	+	2	5.1	393 ± 219.2
P6	+	−	+	+	−	−	1	2.6	497 ± 0.0
P7	+	−	+	+	−	+	6	15.4	855 ± 1167
P8	+	−	+	+	+	+	11	28.2	2028 ± 1756
P9	+	+	+	+	−	+	1	2.6	14,012 ± 0.0
P10	+	+	+	+	+	+	10	25.6	1937 ± 2725
Total ±	34/5	12/27	35/4	36/3	24/15	38/1	39	100	NA

‘+’ represents presence of gene, ‘−’ represents absence of gene, parasite density has been represented as mean ± SD, hrp2F1 and hrp2F2 represent upstream (MAL7P1.230) and downstream (MAL7P1.228) flanking genes of *hrp2* respectively. Similarly, hrp3F1 and hrp3F2 represent upstream (MAL13P1.485) and downstream (MAL13P1.475) flanking genes of *hrp3* respectively

### Parasitaemia

**Fig. 3:**
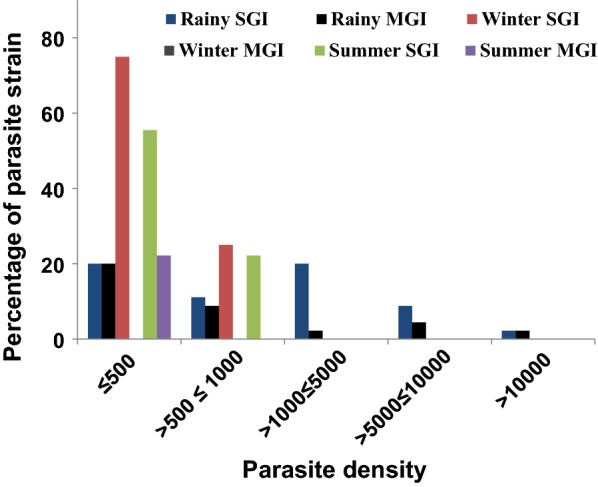
Distribution of parasite strains as SGI or MGI in different seasons at various parasite density. *SGI* single genotype infection, *MGI* multiple genotype infection

Comparison of peripheral blood parasitaemia level between RDT positive (Mean ± SE: 3127 ± 375) and RDT negative samples (1914 ± 434.1) did not reveal any statistical difference. However, among the RDT negative samples, dual negative (1071 ± 519.5) and dual positive (1302 ± 423.1) for *hrp2* and *hrp3* had significantly less parasitaemia level compared to RDT positive samples (P = 0.0033 and P = 0.0041, respectively). Parasitaemia level of samples single negative for either *hrp2* or *hrp3* did not show any difference with RDT positive blood parasitaemia. Of the 9 samples with parasitaemia level recorded below 200 parasites/µl, 7 were observed during off season (6 in summer and 1 in winter). Further, distribution of RDT negative parasite strains as either SGI or MGI in different season showed (Fig. 3) low parasitaemia level in samples (less than 500 parasites/µl) collected during summer and winter with a preponderance of SGI, whereas samples of rainy season were available with multiple infections of parasite for all grades of parasitaemia distribution (as shown in Fig. 3), though the overall frequency of singly infected samples were comparatively more than MGI.

### HRP2 quantification by ELISA

When RDT negative samples were screened for the presence of HRP2 protein using RDT positive sample as positive control in ELISA, except for the control, none of the RDT negative samples had detectable level of the HRP2 antigen.

### Variation in*Pfhrp2* and *Pfhrp3* genes

PCR amplification showed various fragments for *pfhrp2* (600–960 bp) and *pfhrp3* (477–832 bp). In order to examine the repeat sequences in exon2, five samples each positive for *pfhrp2* and for *pfhrp3* among RDT negative samples were randomly selected for sequencing. The parasite strains though exhibited distinct sequences in respect to number, composition and order of repeat types; the repeats type 1 (AHHAHHVAD), type 2 (AHHAHHAAD), type 4 (AHH), type 7 (AHHAAD), and type 12 (AHHAAAHHEAATH) [23, 36] were observed in all isolates. A total of 19 repeats (of which, 9 reported previously and 10 unique repeats) for *pfhrp2* and *pfhrp3* were observed (Table 3). The sequences of *pfhrp2* and *pfhrp3* representing the repeat types were submitted to Gen Bank database (Accession number: MH352454-456).

**Table 3:** Amino acid repeat sequences in *pfhrp2* and *pfhrp3* genes of *P. falciparum* isolates of Odisha

S. n	Repeat	*pfhrp2*	*pfhrp3*
1	AHHAHHVAD	+	+
2	AHHAHHAAD	+	+
3	AHHAHHAAY	−	+
4	AHH	+	+
5	AHHAHHASD	−	−
6	AHHATD	+	−
7	AHHAAD	+	+
8	AHHAAY	+	−
9	AAY	+	−
10	AHHAAAHHATD	−	−
11	AHN	−	−
12	AHHAAAHHEAATH	+	+
Repeat types 13–24 were absent in both *pfhrp2* and *pfhrp3*			
25	AHHAHHAHAAD	+	+
26	AHAHADA	+	−
27	AHHAHHH	+	−
28	AVADAHHHHVADHHAHAAD	+	−
29	AHHGHHAADAHHADSHHAHADAH	+	−
30	AHHDAD	+	−
31	VDD	−	+
32	AHD	−	+
33	AHHATAADAD	−	+
34	AHYAHHAHAAD	−	+

## Discussion

Malaria in Odisha is mostly due to *P. falciparum* infections and in majority of the areas microscopy is inaccessible advocating the use of RDT as the choice for diagnosing malaria in case management [27, 37]. However, substantial evidences of PfHRP2-based RDT failure in diagnosing *P. falciparum* infections in recent years from various parts of South America, Africa and Asia including India [17–19, 21, 30, 38–40, 49] is worrisome. Although false negativity of HRP2 based RDT or RDT failure has been reported from two district of Odisha, India during rainy season [30], its prevalence in other parts of the state and during different seasons are not documented yet. Therefore, the present cross-sectional study was conducted in 25/30 districts of the state representing all four geo-physical regions and in different seasons of the year in order to determine the prevalence of HRP2 based RDT failure. Further, the three major factors (parasite density, *pfhrp2/pfhrp3* gene deletion and polymorphisms of the *pfhrp2/pfhrp3* gene) affecting the performance of PfHRP2-RDT were also examined. The results showed RDT failure in approximately 15% of the microscopically confirmed mono-infected *P. falciparum* samples with high proportions of this false negativity observed in EG and NP region of the state. Previous study conducted in India has reported up to 11% of RDT failure in certain region with EG region of Odisha has RDT failure documented to be 7.8% [30], which is much lower than the present study from the same region (i.e. 20.8%). Since, PfHRP2-RDT is often used as the diagnostic tool for malaria treatment in these areas, failure in RDT and subsequent lack of treatment or improper treatment might have led to rapid selection and transmission of these HRP2 negative parasites. Further, incidence of malaria in EG and NP regions of the state being exceptionally high [28], upon successful treatment of patients carrying RDT sensitive parasites, it is obvious that the proportions of undetected parasites would be comparatively more than other regions of the state as observed in this study. Besides, the involvement of factors affecting RDT performance like age dependent host immunity [41], parasite density [42, 43] or other confounding factors including mosquito preferences to RDT sensitive vs. PfHRP2 negative parasite cannot be ruled out for such large difference (7.8 vs. 20.8) in prevalence of HRP negative parasites. In spite of less number of samples collected during summer and winter (Table 1), the proportions of RDT negative for *P. falciparum* was higher in these periods compared to samples collected during the peak season of transmission in rain. This observation is consistent with the earlier reports of fall of sensitivity of RDT with decrease in transmission intensity [39, 44–46]. Similar to present findings, high prevalence of PfHRP*2*-negative isolates in Mali has been recorded at the end of summer [39]. Assuming that RDT sensitive and RDT negative parasites are transmitted with equal opportunities; *msp1* genotyping was performed to monitor multiplicity of infections only in RDT negative samples. The results showed low multiplicity of infections in RDT negative samples consistent to previous reports [39] and that the frequencies of single strain infections were more in off season compared to peak season of malaria transmission (Fig. 3) which is at par with transmission intensity. Although there was no statistical difference in parasitaemia level between RDT sensitive and RDT negative samples, 7/9 RDT negative *P. falciparum* infected patients with parasitaemia level well below 200 parasites/µl were recorded in low transmission period and that low parasite density at this level (i.e. < 200 parasites/μl) has been shown to affect PfHRP2 based RDT sensitivity [36]. Therefore, the seasonal fluctuations in RDT performance in the present study can be attributed to lack or least mixed infection of PfHRP*2*-negative isolates with RDT sensitive strain in low transmission period undermining the detection of *P. falciparum* infection by RDT, withholding anti-malarial treatment in RDT negative malaria patients leading to rise in transmission of PfHRP2-negative isolates and low parasitaemia level resulting in insufficient production of detectable HRP2 besides other factors.

Genotyping results in RDT negative samples confirmed for the presence of gene deletions and polymorphisms at *hrp2* and *hrp3* genes among natural isolates in symptomatic patients throughout the state at varying proportions. Further, high prevalence of *hrp2* deletion were observed compared to *hrp3* deletion in all four regions. Similar predominant *hrp2* deletion strains have been reported in previous studies from India [30] and Suriname [21], which are in contrast to other studies [17–19, 47, 38–40, 48, 49], where the frequency of *hrp3* deletion was higher. Interestingly, high proportions of *hrp2* deletion parasites were observed during summer, whereas the proportions of *hrp2* deletion and *hrp3* deletion parasites were nearly the same in peak season of malaria transmission (Table 1). Although the reason for such seasonal fluctuation in frequency distribution of *hrp2* and *hrp3* deletion parasite strains is not known, it is expected that more of the detection failure by RDT in low transmission period of summer would be due to *hrp2* deletion because of its high prevalence in the region compared to *hrp3*, reduced co-infection (either with RDT sensitive, *hrp3* negative parasites or both) and successful treatment of patients infected with RDT sensitive parasites or HRP3 negative but HRP2 positive parasites (as HRP2 antigen is the main target for RDT detection) upon detection by RDT. This speculation is further supported by our observation of only SGI in winter, and appearance of MGI (2/9) at low frequency in summer compared to rainy season (17/45) among RDT negative samples despite small sample size. While double negative variants were obtained in 22.4% of RDT negative samples in the present study which is in consistent with the observations from Peru (21.6%) [18]; other studies reported high prevalence [49, 50] or lack of double negative isolates [51]. The overall discrepancy in prevalence of *hrp2* and *hrp3* deletion parasites in different endemic regions could be due to region specific emergence and selection of corresponding deletion variant, transmission intensity and rate of genetic crossing, or geographical spread from neighboring province or countries. Haplotype analysis of singly infected parasites based on presence and absence of *hrp2/hrp3* and their flanking genes revealed 10 different patterns of parasite strain, of which, the four major patterns (Table 2, P1, 7, 8 and 10) were distributed all throughout the state indicating that no specific pattern is confined to any region of the state and the distribution is uniform.

Although low parasitaemia has been shown to influence RDT detection, the observation of gene deletions at *hrp2* and *hrp3* loci in both low and high parasitaemic patient along with no statistical difference at blood parasitaemia level between RDT sensitive and RDT negative isolates suggest that RDT failure in these samples was not due to low parasitaemia rather due to lack of HRP2 protein. This was also evidenced by undetectable PfHRP2 protein in ELISA test. Further, in HRP2 negative isolates (Table 2) confirming the presence of *hrp2/hrp3* and their flanking genes with considerable parasite density, the false negative RDT result could be due to variation in number, composition and types of sequence repeat as observed in this study. Similar variations in PfHRP2 and HRP3 proteins have been shown to influence HRP2 based RDT sensitivity [12, 36] and have been reported from different malaria endemic regions among RDT negative isolates [7, 18, 23, 30, 36]. Altogether, the findings of the present study suggest RDT failure in the study period was largely due to failure of parasite to express the antigen and/or due to alteration of PfHRP2/HRP3 protein sequence affecting the RDT performance. However, the role of low parasitaemia contributing to RDT negativity cannot be ruled out as off season samples with RDT failure mostly had low parasitaemia level. The occurrence and transmission of natural parasite isolates lacking both *hrp2* and *hrp3* though has been reported in several studies [18, 30, 48, 50], the finding of low parasitaemia in these sub-set of patients in the present study suggest for their poor fitness compared to *hrp2* or *hrp3* negative parasites (as failure of producing HRP by one gene could be compensated by another). This assumption needs to be validated experimentally. In absence of definite function, it is difficult to predict the role of HRP in parasite virulence and fitness, however, its presence in all stages of development of parasite [7–9] explain certain survival advantage though not essential for survival. The proposed explanation of detoxification of free haem converting it to haemozoin [52, 53], modulation of infected RBC [54] or host immune response [55] favouring parasite competence to grow with in human host cannot be excluded.

The present study had several limitations. First, the microscopically-confirmed and RDT sensitive *P. falciparum* samples have not been examined for quantitative estimation of HRP2 antigens and genotyping for *hrp2*/*hrp3* deletions or their polymorphisms. The fact that sensitivity of RDT is greatly affected by HRP2 antigen, than parasitaemia [56]; however, correlation of the initial level of blood parasitaemia with HRP2 concentration could not be made. Besides, whether absence of *hrp2* or *hrp3* is compensated by the presence of *hrp3* or *hrp2,* respectively in RDT performance due to cross reaction in this region could not be determined. In such case, mixed infection with sensitive parasites or parasite with alternate *hrp* deletion would underestimate the actual prevalence of *hrp2* and *hrp3* deletions. Moreover, comparison of sequence variation at *hrp2* or *hrp3* between RDT sensitive and RDT negative isolates could have resulted in identifying the region specific potential repeat type, or number of repeats or composition of amino acid sequence as optimal epitopes of RDT performance. Second, only symptomatic patients were screened for malaria diagnosis; however, RDT false negativity has been frequently reported among individuals with low or subpatent level of parasitaemia in asymptomatic patients in some study [39]. As asymptomatic patients serve as the silent reservoir of malaria transmission, presence of HRP2 negative parasites in these patients could be the source of infection to mosquitoes or in blood transfusion for further transmission and may become an obstruction to elimination efforts of malaria. After all, the strength of the present study was that the natural *P. falciparum* isolates from all four geo-physical regions of the state were screened for the prevalence of *hrp* negative parasites. Besides, the study was conducted in three different seasons and *hrp* negative isolates were detected in all throughout the year which is a major threat to malaria control programmes, if HRP2-based RDT becomes the choice of malaria diagnosis for case management.

## Conclusion

The findings of the study reveal prevalence of HRP2 based RDT negative *P. falciparum* among natural isolates of Odisha, India in all four geo-physical regions of the state and in different seasons. The failure of RDT in detecting *P. falciparum* infection was due to undetectable level of HRP2 antigen. The observed lack of sensitivity in RDT has been explained by gene deletions and/or genetic diversity at *hrp2* and *hrp3* loci though low level of parasitaemia could have reduced the sensitivity further in some cases. High prevalence of parasites with *hrp2* deletion including dual deletions (*pfhrp2* and *pfhrp3*) is a serious cause of concern as these patients could not be given a correct diagnosis and treatment. Therefore, HRP2-based RDT for diagnosing *P. falciparum* infection in Odisha is non-reliable and must be performed in addition to or replaced by other appropriate diagnostic tools clinical management of the disease.

## Additional file

**Additional file 1: Table S1.** Primers and the reaction conditions.
