# Supplementary material for: High proportions of pfhrp2 gene deletion and performance of HRP2-based rapid diagnostic test in Plasmodium falciparum field isolates of Odisha
Source: Malar J. 2018 Oct 29;17:394. doi: 10.1186/s12936-018-2502-3 (PMC6206925; doi:10.1186/s12936-018-2502-3)
Supplement: Supplementary file 1 — Additional file 1: Table S1. Primers and the reaction conditions. [file 12936_2018_2502_MOESM1_ESM.doc]

**Table S1: Primers and the reaction conditions**

| **Gene Primer Sequence** |  | **programme** |
| --- | --- | --- |
| **MSP1 (Primary)** | **F: CACAATGTGTAACACATGAAAG**  **R: AGTACGTCTAATTCATTTGCAC** | **94°C 1 Min 55°C 1 Min**  **72°C 1 Min 35cycle** |
| **MSP1 (Nested)** | **F: CACAATGTGTAACACATGAAAG**  **R: AGTACGTCTAATTCATTTGCAC** | **94°C 1 Min 53°C 1 Min**  **72°C 1 Min 25 cycle** |
| **MSP2 (Primary)** | **F: ATGAAGGTAATTAAAACATTGTC**  **R: TTATTGAAGCAATATTACTAGAG** | **94°C 1 Min 53°C 1 Min**  **72°C 1 Min 35 cycle** |
| **MSP2 (Nested)** | **F: AGCAACACATTCATAAACAATG**  **R: CACAGTTTTCTTTGTTACCATC** | **94°C 1 Min 54°C 1 Min**  **72°C 1 Min 25cycle** |
| **Flanking genes** |  |  |
| **Pfhrp2 Upstream PF3D7_0831900) (Primary)** | **F: GATATCATTAGAAAACAAGAGCTTAG**  **R: TATCCAATCCTTCCTTTGCAACACC** | **94°C 1 Min 63°C 1 Min 72°C 1 Min 35** |
| **PF3D7_0831900 (MAL7P1.230) (Nested)** | **F: TATGAACGCAATTTAAGTGAGGCAG**  **R: TATCCAATCCTTCCTTTGCAACACC** | **94°C 1 Min 65°C 1 Min 72°C 1 Min 25 cycle** |
| **PfHRP2-2** | **F: CAAAAGGACTTAATTTAAATAAGAG**  **R: AATAAATTTAATGGCGTAGGCA** | **94°C 1 Min 55°C 1 Min 72°C 1 Min 35 cycle** |
| **Pfhrp2 Downstream PF3D7_0831700 (MAL7P1.228) (Primary)** | **F:AGACAAGCTACCAAAGATGCAGGTG**  **R: TAAATGTGTATCTCCTGAGGTAGC** | **94°C 1 Min 60°C 1 Min 72°C 1 Min 35 cycle** |
| **PF3D7_0831700 (MAL7P1.228) (Nested)** | **F: CCATTGCTGGTTTAAATGTTTTAAG**  **R: TAAATGTGTATCTCCTGAGGTAGC** | **94°C 1 Min 63°C 1 Min 72°C 1 Min 25 cycle** |
| **Pfhrp3 Upstream PF3D7_1372100(MAL13P1.485) (Primary)** | **F: TTGAGTGCAATGATGAGTGGAG**  **R: AAATCATTTCCTTTTACACTAGTGC** | **94°C 1 Min 60°C 1 Min 72°C 1 Min 35 cycle** |
| **PF3D7_1372100(MAL13P1.485) (Nested)** | **F: GTTACTACATTAGTGATGCATTC**  **R: AAATCATTTCCTTTTACACTAGTGC** | **94°C 1 Min 59°C 1 Min 72°C 1 Min 25 cycle** |
| **PfHRP3-2** | **F: AATGCAAAAGGACTTAATTC**  **R: TGGTGTAAGTGATGCGTAGT** | **94°C 1 Min 55°C 1 Min 72°C 1 Min 35 cycle** |
| **Pfhrp3 downstream PF3D7_1372400 (MAL13P1.475) (Primary)** | **F: TTCATGAGTAGATGTCCTAGGAG**  **R: TCGTACAATTCATCATACTCACC** | **94°C 1 Min 55°C 1 Min 72°C 1 Min 35 cycle** |
| **PF3D7_1372400 (MAL13P1.475) (Nested)** | **F: TTCATGAGTAGATGTCCTAGGAG**  **R: GGATGTTTCGACATTTTCGTCG** | **94°C 1 Min 61°C 1 Min 72°C 1 Min 25 cycle** |
| **Pf HRP2 Additional primer** | **F-TGTGTAGCAAAAATGCAAAAGG**  **R-TTAATGGCGTAGGCAATGTG** | **94°C 30sec 57°C 40sec 72°C 90sec 35 cycle** |
| **Pf HRP3 Additional primer** | **F- AAATAAGAGATTATTACACGAAAG**  **R- TGGTGTAAGTGATGCGTAGT** | **94°C 30sec 57°C 40sec 72°C 90sec 35 cycle** |
